# Feasibility and acceptability of Nenne Navi-AI: family-tailored intervention to improve sleep in young Japanese children

**DOI:** 10.3389/frsle.2026.1827400

**Published:** 2026-06-23

**Authors:** Arika Yoshizaki, Manabu Saito, Ai Terui, Kanako Kawamura, Emi Murata, Sanae Tanaka, Ikuko Hirata, Ikuko Mohri, Kazunori Komatani, Masako Taniike

**Affiliations:** 1 Molecular Research Center for Children's Mental Development, United Graduate School of Child Development, The University of Osaka, Suita/Osaka, Japan; 2 Department of Clinical Psychological Science, Graduate School of Health Sciences, Hirosaki University, Hirosaki/Aomori, Japan; 3 Department of Neuropsychiatry, Graduate School of Medicine, Hirosaki University, Hirosaki/Aomori, Japan; 4 Department of Child Development, United Graduate School of Child Development, The University of Osaka, Suita/Osaka, Japan; 5 Research Center for Child Mental Development, Kanazawa University, Kanazawa/Ishikawa, Japan; 6 SANKEN, The University of Osaka, Ibaraki/Osaka, Japan

**Keywords:** artificial intelligence (AI), behavioral intervention, digital health intervention, feasibility study, pediatric sleep, personalized sleep intervention, sleep habits

## Abstract

**Introduction:**

Despite advancements in sleep medicine, inadequate sleep habits among young children persist. Establishing appropriate sleep habits in early childhood is essential for supporting physical, emotional, and cognitive development. However, scalable and personalized behavioral interventions for caregivers in community settings remain scarce, particularly AI-enabled systems designed for real-world implementation.

**Methods:**

This study evaluated adherence, perceived usefulness, and feasibility of Nenne Navi-AI among 50 caregivers recruited in Hirosaki City, Japan, through community health checkups, childcare facilities, and public advertisements. The culturally tailored application integrates supervised machine-learning models with rule-based algorithms to provide personalized guidance and ongoing support for promoting healthier sleep habits.

**Results:**

During the 6-month intervention, only 3 of 50 caregivers (6%) experienced continuous 3-month data-entry lapses, with no withdrawals. Significant pre-post improvements were observed in children's number of awakenings after sleep onset and subjective sleep quality ratings. Subgroup analyses suggested improvements among children with poorer baseline sleep habits (≥0.5 SD worse than the sample mean). Post-intervention assessments confirmed high caregiver acceptability, satisfaction, and reduced parenting stress.

**Conclusions:**

Nenne Navi-AI demonstrates high feasibility with excellent 6-month adherence and favorable usability feedback. The system shows promise for improving early childhood sleep (night-waking), enhances caregiving experiences, reduces negative parenting emotions, and provides a scalable framework for future AI-enabled pediatric sleep interventions.

## Introduction

Growing evidence from cohort studies and reviews indicates that healthy sleep during early childhood is associated with better physical and mental health development (Sivertsen et al., 2015; Matthews and Pantesco, 2016; Bathory and Tomopoulos, 2017; Huhdanpää et al., 2019; Morales-Muñoz et al., 2020; Sivertsen et al., 2021; Lokhandwala and Spencer, 2022; Liu et al., 2024; Finkel et al., 2024). Young children in Japan exhibit relatively short nocturnal sleep duration compared with recommended values and international norms (Mindell et al., 2010; Murata et al., 2023; Su et al., 2024; Wang et al., 2024). Recent population-based studies in Japan have also reported a substantial prevalence of sleep problems among children (Kuki et al., 2024). Kohyama et al. (2002) demonstrated a nationwide trend of delayed bedtimes among 3-year-old children in Japan. Recent studies report prolonged screen time in urban areas, underscoring the impact of the living environment on children's sleep habits (Sato and Kanikowska, 2025).

In-person sleep interventions often require substantial time commitments, specialized personnel, and sustained participant engagement, presenting barriers to widespread implementation in routine care. Given these constraints, digital health interventions have gained attention as flexible and scalable solutions for delivering pediatric sleep support across diverse settings. A recent scoping review suggests that mobile applications and digital interventions may support sleep health management among adolescents and adults (Al Mahmud et al., 2022). However, most existing digital sleep tools rarely focus on infants and toddlers, despite the distinct caregiving demands of early childhood. Moreover, despite the importance of these factors in supporting caregivers of young children, many existing digital sleep tools provide limited personalization and lack adequate clinical validation and contextual adaptation (Ananth, 2021; Lu et al., 2025). Mindell et al. (2011) and Leichman et al. (2020) demonstrated the efficacy of internet- and mHealth (mobile health)-based behavioral interventions for improving infant and toddler sleep habits. Early intervention targeting both sleep and crying problems has shown effectiveness in preventing postnatal depression (Hiscock et al., 2014). Implementing these approaches in Japan requires careful consideration of the country's predominant co-sleeping practices and family contexts, which may influence caregiver engagement and the applicability of standardized behavioral recommendations.

To address these challenges, the authors developed Nenne Navi^®^ (meaning “sleep navigator” in Japanese), an interactive and personalized digital intervention tailored to Japanese cultural practices, family rhythms, and parent-child interactions. The effectiveness of this human-operated app version has been confirmed in a 1-year single-site intervention trial (Yoshizaki et al., 2023) and a 6-month multi-site intervention trial (Tanaka et al., 2025), establishing both clinical efficacy and implementation feasibility across community settings.

While social implementations in Nenne Navi^®^ involved direct guidance from sleep experts via the app, recent advances in artificial intelligence (AI) and machine learning (ML) now enable automated adaptive behavioral support systems (Jiang et al., 2017; An et al., 2023). AI-supported interventions can analyze user inputs, deliver personalized recommendations, sustain engagement, and provide timely guidance without ongoing expert involvement (Verma et al., 2023; Gkintoni et al., 2025). Integrating expert knowledge with AI-based inference may further enhance the safety, clinical relevance, and scalability of digital behavioral interventions. A scoping review suggests that mHealth apps for caregiver decision-making in child health are generally useful and acceptable; however, rigorous evaluations of personalized digital guidance in real-world pediatric sleep contexts remain scarce (Dai et al., 2026). Recent studies have underscored key limitations of data-driven ML approaches in clinical decision support, including limited transparency, insufficient explainability, and poor adaptability to domain-specific requirements (Holzinger et al., 2017; Kierner et al., 2025). Hybrid architectures that combine ML with rule-based expert knowledge have emerged as a promising approach for clinical decision support, offering scalable performance while preserving interpretability and contextual responsiveness (Kierner et al., 2025; Yimenu et al., 2025). In early childhood sleep support, such hybrid systems are particularly advantageous because they can incorporate family routines, cultural norms, and caregiver preferences—factors that data-driven models often struggle to capture due to their nuanced and context-dependent nature.

Building on this foundation, we developed “Nenne Navi-AI,” an enhanced bidirectional smartphone app that automates the selection and tailoring of personalized recommendations using supervised machine learning models and rule-based algorithms. These recommendations align with each household's daily routines and schedules (e.g., sleep–wake patterns, daytime activities, mealtimes, screen time, and bedtime routines), as well as cultural norms and parenting challenges, thereby reducing reliance on the manual expert review required in the original version.

Given the novelty of this AI-enhanced intervention and the need to assess its utility and engagement in community settings, we conducted an initial feasibility study prior to large-scale effectiveness trials.

The primary outcome of this feasibility study was adherence to the intervention, assessed by measuring dropout rates and app engagement over 6 months. The secondary outcome was preliminary improvement in children's sleep habits. Exploratory outcomes included caregiver acceptability, perceived usefulness, satisfaction, and perceived changes in caregiving practices.

## Materials and methods

### Study design

A single-arm pilot trial of Nenne Navi-AI was conducted in Hirosaki City, Japan to evaluate the feasibility of the AI-enhanced intervention in a real-world community setting. Participants were recruited between September 2022 and December 2024 through an ongoing community-based enrollment until the target sample size (*n* = 50) was reached. Each participant used the Nenne Navi-AI intervention for a 6-month period. Follow-up assessments, including online surveys and interviews, were conducted at the end of the 6-month intervention period. The system remained operational until June 2025, and follow-up interviews were completed in July 2025.

The Nenne Navi-AI intervention was delivered via the LINE platform, widely used in Japan. Participants accessed the AI-enhanced sleep intervention by adding the Nenne Navi-AI LINE account as a friend on their mobile devices. The intervention was implemented as a LINE-based application connected to a backend AI system. Messages and notifications, including supportive messages and prompts for data entry or advice selection, were delivered through the LINE platform. The app's usability and effectiveness were evaluated using data on sleep-related habits recorded in the app, post-intervention online surveys, and interviews conducted following completion of the 6-month intervention.

### Study site

Hirosaki City (Aomori Prefecture, northern Japan; ~160,000 population) served as the primary trial site. The city maintains a structured maternal-child health system. Hirosaki University is conducting an ongoing community-linked developmental cohort study, including online developmental screening at ages 3 and 5 years in cooperation with Hirosaki City. Hirosaki previously served as a trial site for Nenne Navi^®^ (Yoshizaki et al., 2023), providing relevant experience in community implementation.

### Participants

Participants were recruited from the community in Hirosaki City, Japan, during health checkups for 1.5-year-olds, local childcare support facilities, newspapers, and social networking services between September 2022 and December 2024. Caregivers of children aged 1.5–3 years with the following sleep problems were recruited: (1) falling asleep after 10 pm, (2) getting < 9 h of nighttime sleep, (3) frequent nighttime awakenings, and (4) irregular sleep–wake rhythms. In this study, no formal screening procedures or eligibility criteria regarding the presence, duration, or severity of sleep problems were applied, and caregivers were not required to report persistent sleep problems for any specific minimum duration (e.g., 3 months) prior to enrollment. Caregivers could also participate if they desired guidance on their child's sleep, regardless of whether these issues were present.

Eligible participants were caregivers who owned an internet-enabled mobile device (iOS or Android), agreed to add the Nenne Navi-AI LINE account as a friend, and could use the app and enter data in Japanese. Because the intervention was designed to support healthy sleep habits in community settings, caregivers were eligible regardless of whether their children had sleep problems. All enrolled participants reported their children's sleep habits (e.g., bedtime, wake-up time, night awakenings, and daytime activities) and subjective sleep quality, as well as their own sleep habits and subjective sleep quality, using the app over a 6-month period [see Multimedia Appendix of Yoshizaki et al. (2020) for detailed items]. Written informed consent was obtained from all participants. After the intervention period, all enrolled participants were invited to complete an online questionnaire and participate in semi-structured online interviews as part of the post-intervention user experience assessment.

### Intervention: Nenne Navi-AI system

**Figure 1:**
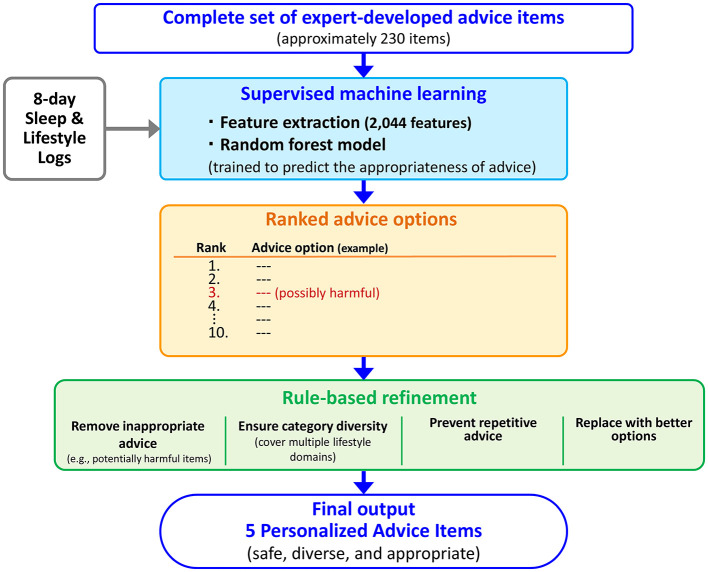
Overview of the personalized advice selection process in Nenne Navi-AI.

Nenne Navi-AI is a mobile-based behavioral sleep support system designed to help caregivers improve their children's sleep habits through personalized recommendations and ongoing feedback. The overall structure and core functions of the original Nenne Navi^®^ app are described in detail in other sources (Yoshizaki et al., 2020, 2023; Tanaka et al., 2025). In brief, the system integrates daily routine data (e.g., wake/bedtimes, naps, activities, media use, meals, and co-sleeping) to provide specific, small-step recommendations (e.g., “try finishing naps by 3:00 p.m.”). Caregivers select monthly goals, report adherence, and receive automated encouraging feedback messages based on expert-developed templates, with progress visualized through graphs and gamified elements. In the Nenne Navi^®^ system, caregivers record their children's sleep habits for eight consecutive days each month. Based on these records, pediatric sleep experts manually select five tailored pieces of advice, from which caregivers choose one to implement during the following month. In Nenne Navi-AI, this advice selection process is supported by an AI-based algorithm that selects and tailors personalized recommendations. As shown in Figure 1, the caregivers received multiple personalized sleep advice items selected for implementation during the subsequent period.

The AI technology was conceptualized by the research team and integrated into the intervention system with support from the IT company that developed the application (Panasonic Advanced Technology Development Co., Ltd., Osaka, Japan) under the supervision of K.K., a professor at The University of Osaka with expertise in AI. For the AI component, expert-developed advice served as ground-truth labels within a supervised machine learning framework.

#### Hybrid system development and iterative refinement

To construct the training dataset, weekly sleep-habit data were collected from 4,912 toddlers aged 18–30 months via a web-based survey. Cases were excluded if they lacked four consecutive days of sleep habit data, had incomplete responses or implausible values (e.g., reported wake-up times later than nap times), or if the respondent was not the mother. This restriction was applied to ensure consistency in caregiver-reported sleep routines, as mothers were the primary respondents in the survey and the main caregivers responsible for recording children's daily sleep habits. After data cleaning, the final analytic sample comprised 2,155 cases. Of these, 737 cases were used as the training dataset for machine learning, and the remaining 1,418 cases served as the test dataset.

The training dataset comprised caregiver-reported sleep and lifestyle logs collected over 8 consecutive days (approximately 30 sleep- and lifestyle-related items per day), paired with five expert-developed personalized advice items for each case, which served as the training labels.

Training labels were created by a team of pediatric sleep medicine experts, including three pediatricians and three psychologists. To minimize qualitative bias in expert-developed advice, each piece of advice was collaboratively developed by a pair of a pediatrician and a psychologist.

To provide personalized advice framed as achievable small-step goals tailored to each child's current sleep habits, a hybrid approach combining a random forest model with rule-based expert algorithms was employed. The random forest model was trained on 2,044 engineered features derived from behavioral, environmental, and family-related inputs, including key variables such as the child's age in months, sleep location, co-sleeping status, daytime childcare arrangements, breastfeeding status, availability of caregiving support, primary bedtime caregiver, bedtime and wake-up times, sleep onset latency, nighttime sleep duration, and lifestyle-related factors such as evening routines, physical activity, and media use. These features were generated through data processing of the original survey variables, including transformation of sleep timing variables, encoding of categorical responses, and derivation of behavioral indicators reflecting daily sleep routines and family context.

The personalized advice system was developed through two sequential beta versions of a hybrid architecture combining machine learning-derived advice with expert-designed rule-based algorithms.

#### Beta version 1: initial hybrid model

**Figure 2:**
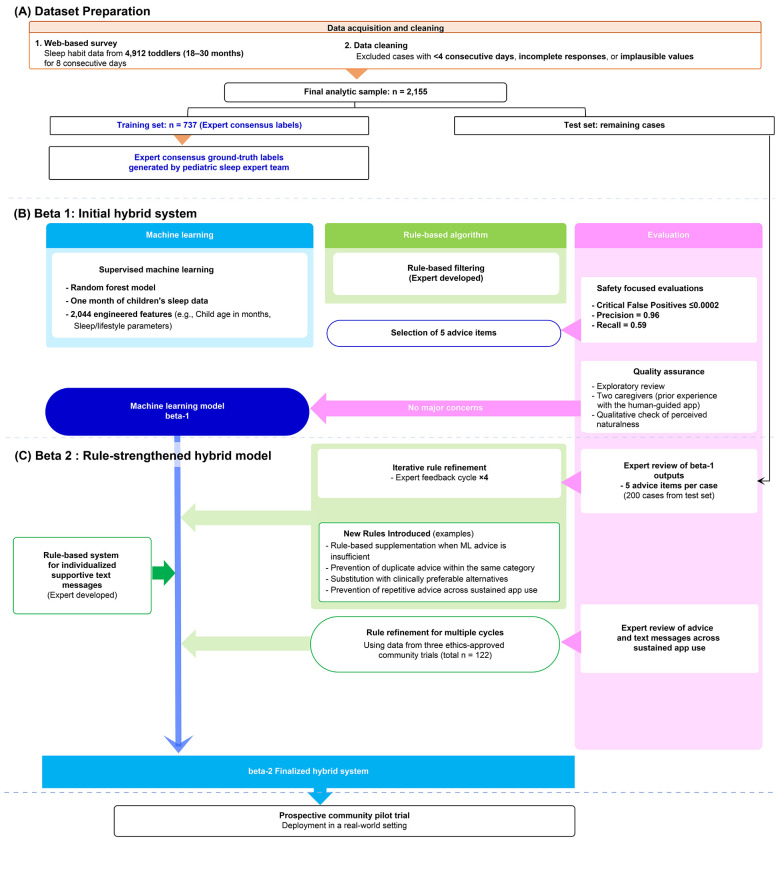
The development process consisted of three stages: **(A)** dataset preparation for supervised machine learning. **(B)** machine learning training and initial hybrid system development with safety validation (Beta 1), and **(C)** rule strengthening through expert review of model interface outputs without retraining (Beta 2).

An initial hybrid system was constructed by integrating machine-learning-inferred advice with rule-based filtering using children's sleep data collected over a single monthly cycle (8 consecutive days per month). Specifically, the final five advice items delivered via the app were selected from machine-learning-ranked candidates using an expert-designed filtering system (e.g., ensuring diversity across advice categories such as wake-up time, bedtime, and outdoor activity). The complete set of expert-developed advice items comprised approximately 230 items, forming the candidate pool for model-based ranking. An overview of the system development process is shown in Figure 2.

Performance was evaluated with primary emphasis on safety, given the preventive nature of the intervention and its pediatric target population, consistent with prior evidence highlighting the importance of patient safety and harm avoidance in pediatric and preventive digital health interventions (Park et al., 2024). Model performance metrics (precision and recall) were calculated on the held-out test dataset. Potentially harmful advice was defined a priori as critical false-positive advice (e.g., recommendations that may worsen sleep, such as later bedtimes, excessive or late screen times, or more burdensome bedtime practices), with minimizing its occurrence set as a key evaluation objective. The beta-1 model achieved a critical false-positive rate of ≤ 0.0002, satisfying the primary safety criterion. Additionally, precision was targeted at ≥0.95 to ensure that, across four advice sessions (each providing five recommendations), no more than one recommendation would differ from those provided by human sleep experts. Recall was targeted at ≥0.60 (i.e., ≥3 of 5 recommendations consistent with those of experts). The beta-1 model achieved a precision of 0.96 and a recall of 0.59, indicating that the predefined performance targets were largely met. These metrics supported the acceptability of the system for safety-prioritized, community-based use.

As part of quality assurance, a preliminary qualitative assessment of perceived naturalness was conducted prior to final rule refinement. Two caregivers who received a human-guided Nenne Navi^®^ version evaluated advice and messages in an unblinded, exploratory manner. Given the small sample size, this assessment was intended only as an exploratory quality check and does not provide representative evidence of usability. No major concerns regarding user disengagement were identified.

#### Beta version 2: rule-strengthened hybrid model

The beta 2 system strengthened rule-based components to minimize false positives and negatives. As part of this refinement process, the expert team reviewed the advice provided by the hybrid system for 200 cases from the web-based survey. For each case, the model selected three to five advice items, which were evaluated for clinical appropriateness. Advice was considered appropriate if it was suitable for the child's reported sleep habits and family context, developmentally suitable for the child's age, consistent with established pediatric sleep guidelines, and unlikely to introduce sleep disruption or safety concerns. Algorithms for advice selection and tailoring were refined iteratively through four rounds of expert feedback.

New rules introduced during this phase included: (1) rule-based supplementation when machine learning selected an insufficient number of advice items, (2) prevention of duplicate advice within the same category, and (3) substitution with clinically preferable alternatives (e.g., weaning guidance tailored to caregiver intentions), among others.

Using de-identified data from three ethics-approved community trials (total *n* = 122), rules were strengthened for sustained app users to prevent repetition of identical advice (e.g., avoiding duplicate advice across consecutive months). Concurrently, pediatric sleep experts developed and refined a separate rule-based system to provide individualized, encouraging feedback messages based on expert-developed message templates for each advice item.

### Sleep parameters

Sleep parameters were collected monthly over 8 consecutive days from pre-intervention to 6-month post-intervention. All sleep parameters were recorded by caregivers through daily logs entered within the mobile application. In addition to standard metrics (wake-up time, bedtime, sleep onset latency, nighttime sleep duration, with means and SDs), we captured daily habits including naps, evening meals, bathing, physical activity, media use, and other pre-bedtime behaviors. Social jetlag and social sleep restriction were calculated as the midpoint difference and weekend-minus-weekday nighttime sleep duration, respectively. Moreover, caregivers were asked to report their own emotional experiences during daily parenting (e.g., irritability and fatigue) using a simple checklist in the daily logs. These self-reported items were used to derive caregiver-related outcomes, such as the proportion of days with reported irritability. These data guided the selection of recommendations.

### Post-intervention survey and user experience assessment

User experience was assessed using two methods: (1) an online questionnaire administered post-intervention, and (2) semi-structured individual online interviews with consenting participants. The interview protocol included both rating-scale questions (using a five-point Likert scale) and open-ended questions that allowed participants to elaborate on their experiences with the app.

The online questionnaire evaluated usefulness, usability, perceived changes in children's sleep, changes in caregiver self-efficacy, and additional factors. Usefulness items were rated on a five-point Likert scale (1 = “not helpful,” 2 = “somewhat unhelpful,” 3 = “neutral,” 4 = “somewhat helpful,” 5 = “helpful”). Perceived changes in children's sleep were rated similarly (1 = “worsened,” 2 = “somewhat worsened,” 3 = “no change,” 4 = “somewhat improved,” 5 = “improved”). Changes in caregiver self-efficacy for managing their child's sleep and overall parenting were each assessed using five-point self-report scales (1 = “worsened,” 2 = “somewhat worsened,” 3 = “no change,” 4 = “somewhat improved,” 5 = “improved”). Other usability items and aspects were assessed using five-point Likert scales. Quantitative scores were analyzed using descriptive statistics and paired *t*-tests. The questionnaire items for this study were developed based on items used in previous evaluations of the Nenne Navi^®^ system (Yoshizaki et al., 2023; Tanaka et al., 2025) and were not derived from formally validated instruments. Each outcome was assessed using a single item; therefore, composite scores were not calculated.

Semi-structured interviews were conducted based on responses to the online questionnaire, primarily to elicit more detailed, concrete explanations for caregivers' Likert-type ratings of the feasibility and acceptability of the intervention and to further explore caregivers' experiences and perceptions of the AI-enhanced intervention. Specific questions included:—“Did you receive advice that was appropriate for the information you entered?” (rated on a five-point scale: 1 = “often inappropriate,” 2 = “sometimes inappropriate,” 3 = “neutral,” 4 = “mostly appropriate,” 5 = “always appropriate”), and—“How human-like were the advice and comments?” (rated on a five-point scale: 1 = “very mechanical,” 2 = “somewhat mechanical,” 3 = “neutral,” 4 = “somewhat human-like,” 5 = “very human-like”). Interviews were audio-recorded, transcribed, and analyzed descriptively to identify common themes.

### Statistical analyses

Paired *t*-tests were used to examine within-subject differences in children's sleep habits between pre-intervention and post-intervention. Descriptive statistics are reported as means and standard deviations (SD). Similar paired *t*-tests were conducted to assess changes in caregivers' sleep habits. Results are reported as mean differences with corresponding *p*-values. Normality of the paired differences was assessed prior to analysis. When the assumption of normality was not met, results were confirmed using the Wilcoxon signed-rank test, which revealed similar results.

Adherence to the intervention was evaluated based on the continuity of caregivers' data entry during the 6-month intervention period. Consistent adherence was defined as having no more than two consecutive months without any data entry.

Participants were included in the final analyses if they provided sufficient data during the post-intervention assessment period (sixth or seventh cycle). Conversely, those who did not provide sufficient data during this assessment period or failed to complete at least 2 consecutive days of data entry (weekday and weekend) were excluded from the analyses.

Prespecified subgroup analyses examined whether intervention effects differed by pre-intervention sleep habit severity. Children with pre-intervention sleep parameters more than 0.5 SD from the overall mean in an unfavorable direction (i.e., wake-up time or bedtime more than 0.5 SD later than the mean, nighttime sleep duration more than 0.5 SD shorter than the mean, or number of nighttime awakenings more than 0.5 SD higher than the mean) were classified as having poorer pre-intervention sleep habits and analyzed separately. Distribution-based approaches commonly interpret approximately half a standard deviation as a meaningful deviation in health-related outcomes (Norman et al., 2003; Revicki et al., 2008), and a difference of approximately 0.5 SD is generally considered a moderate effect size (Cohen, 1988).

All statistical analyses were conducted using JMP student edition version [19.0.5] for macOS (SAS Institute Inc., Cary, NC, USA). A two-sided *p-*value of < 0.05 was considered statistically significant.

### Ethics statement

This study was approved by the University of Osaka Clinical Research Review Board (CRB5180007) on August 17, 2020, prior to its initiation [No. 22100 (T1)]. All procedures adhered to the ethical standards of the Declaration of Helsinki. At the start of the pre-intervention assessment, participants received detailed information about the study's goals, procedures, and data protection measures. Written consent was obtained from all participants individually before enrollment. Each participant received a coupon for books valued at JPY 5,000 (USD 32). This study was registered with the University Hospital Medical Information Network Clinical Trials Registry (UMIN-CTR), Japan, before the enrollment of the first participant (registration number: UMIN000048683).

## Results

### Demographic information of the study participants

In total, 50 caregiver–child pairs participated in the study. Data from 38 caregiver–child pairs were included in the final analyses after excluding 12 pairs due to insufficient data entry for the post-intervention assessment period. Of those 12, 10 caregivers had no data entry for the sixth or seventh cycle (the scheduled post-intervention assessment period), and 2 additional caregivers did not complete at least 2 consecutive days (weekday and weekend) of data entry during the sixth or seventh cycle. The demographic characteristics of the 38 participants are presented in Table 1. All caregivers included in the final analysis reported at least one sleep-related concern at baseline, including mild concerns.

**Table 1:** Demographic characteristics of the 38 caregiver–child pairs included in the final analytic sample.

Characteristic	Mean (SD)
	*n* = 38
Mothers' age	35.32 (4.67)
Fathers' age	37.46 (6.14)
Children's age in months	21.95 (4.27)
Gender: male/female	22/16
Fathers' education	
Junior high school	0
High school	12
College/University	23
Graduate school	2
Others/n.a.	1
Mothers' education	
Junior high school	0
High school	8
College/University	27
Graduate school	3
Others/n.a.	0
Family income (JPY)	
< 3,000,000	2
3,000,000–5,000,000	7
5,000,000–7,000,000	16
7,000,000–10,000,000	7
>10,000,000	3
Not available	3
Family characteristics	
Siblings: Yes/No	15/23
Nursery school/family care	29/9
Mothers' employment: Yes/No	30/8
Nuclear family/multi-generational family	33/5

### Adherence and retention

During the 6-month intervention, 3/50 caregivers (6%) had ≥3 consecutive data-entry lapses. No participants formally withdrew from the study during the intervention period.

### Data exclusion criteria

Using caregiver-rated five-point scores of children's health condition (ranging from “good” to “poor”) and activity records, daily records were analyzed after excluding days with:

(1) family travel during the period;
(2) child illness, indicated by either caregiver-rated health condition or activity records, including: (a) caregiver-rated poor health condition; (b) preschool/nursery absence due to illness; (c) fever (for children not enrolled in preschool/nursery) accompanied by a caregiver-rated health condition of “somewhat poor” or worse.

After applying these day-level exclusion criteria, participants were included in the longitudinal analyses if sufficient data remained during the post-intervention assessment period (i.e., the 6th or 7th monthly cycle), defined as at least 2 consecutive days of data entry, covering both weekdays and weekends.

### Sleep outcomes of the children

As shown in Table 2, significant pre–post intervention differences were observed in the number of awakenings after sleep onset [pre: 1.51 ± 1.66, post: 0.74 ± 1.23, *t*_(37)_ = 3.34, and *p* = 0.002], nap onset time [pre: 12:48 ± 0:46, post: 13:04 ± 0:39, *t*_(37)_ = 2.61, and *p* = 0.013], and total sleep duration [pre: 11:20 ± 0:39, post: 11:04 ± 0:38, *t*_(37)_ = −2.64, and *p* = 0.012]. Most children had a single daytime nap per day (pre-: mean number of naps = 1.02, SD = 0.28; post-: mean number of naps = 0.96, SD = 0.29), though some variability was observed. Caregivers' ratings of children's subjective sleep quality increased from pre- to post-intervention, although the change did not reach statistical significance [pre: 3.65 ± 0.87, post: 3.93 ± 0.89, *t*_(37)_ = 1.93, *p* = 0.061].

**Table 2:** Sleep parameters of children at pre-intervention and post-intervention (*n* = 38).

	Pre	Post	*p*-value
Measure	Mean ±SD	Mean ±SD	
Wake-up time (hh:mm)	6:55 a.m. ± 0:34	6:48 a.m. ± 0:38	0.222
Wake-up time SD (minutes)	22.93 ± 10.65	21.74 ± 14.88	0.606
Bedtime (hh:mm)	8:57 p.m. ± 0:39	9:01 p.m. ± 0:41	0.504
Bedtime SD (minutes)	23.01 ± 14.95	20.59 ± 17.33	0.503
Sleep onset latency (minutes)	28.00 ± 16.96	25.82 ± 18.22	0.568
Sleep onset latency SD (minutes)	13.96 ± 12.41	11.26 ± 9.99	0.205
Nocturnal sleep duration (hours)	9.48 ± 0.70	9.28 ± 0.62	0.057
Nocturnal sleep duration SD (minutes)	30.06 ± 12.27	30.27 ± 18.48	0.950
Number of awakenings after sleep onset	1.51 ± 1.66	0.74 ± 1.23	0.002
Social jet lag (minutes)	10.05 ± 28.57	5.05 ± 26.22	0.338
Social sleep restriction (minutes)	12.08 ± 38.47	9.84 ± 40.67	0.743
Nap starting time (hh:mm)	12:48 p.m. ± 0:46	1:04 p.m. ± 0:39	0.013
Nap starting time SD (minutes)	49.90 ± 39.23	38.36 ± 36.58	0.100
Nap ending time (hh:mm)	2:48 p.m. ± 0:50	2:59 p.m. ± 0:35	0.190
Nap ending time SD (minutes)	52.58 ± 36.03	40.08 ± 37.37	0.113
Nap duration (hours)	1.86 ± 0.60	1.78 ± 0.60	0.324
Nap duration SD (minutes)	28.72 ± 15.77	25.90 ± 20.12	0.405
Total sleep duration (hours)	11.34 ± 0.66	11:08 ± 0.64	0.012
Total sleep duration SD (minutes)	37.53 ± 12.59	36.98 ± 18.62	0.841
Subjective sleep quality (points)	3.65 ± 0.87	3.93 ± 0.89	0.061
Television-viewing duration (minutes)	90.47 ± 62.33	80.79 ± 62.81	0.301
End of television-viewing time after 4:00 p.m. (hh:mm)	7:52 p.m. ± 0:58	7:52 p.m. ± 1:08	0.505
Smartphone-use duration (minutes)	13.16 ± 19.33	21.92 ± 35.36	0.095
End of smartphone-use time (hh:mm)	6:25 p.m. ± 1:52	5:57 p.m. ± 2:16	0.651
Media use duration (minutes)	103.63 ± 65.80	102.71 ± 79.30	0.932
End of media use time (hh:mm)	7:56 p.m. ± 0:55	7:55 p.m. ± 1:05	0.442

Differences between pre-intervention and post were tested using the paired t-test.

### Subgroup analyses of children's sleep outcomes

**Figure 3:**
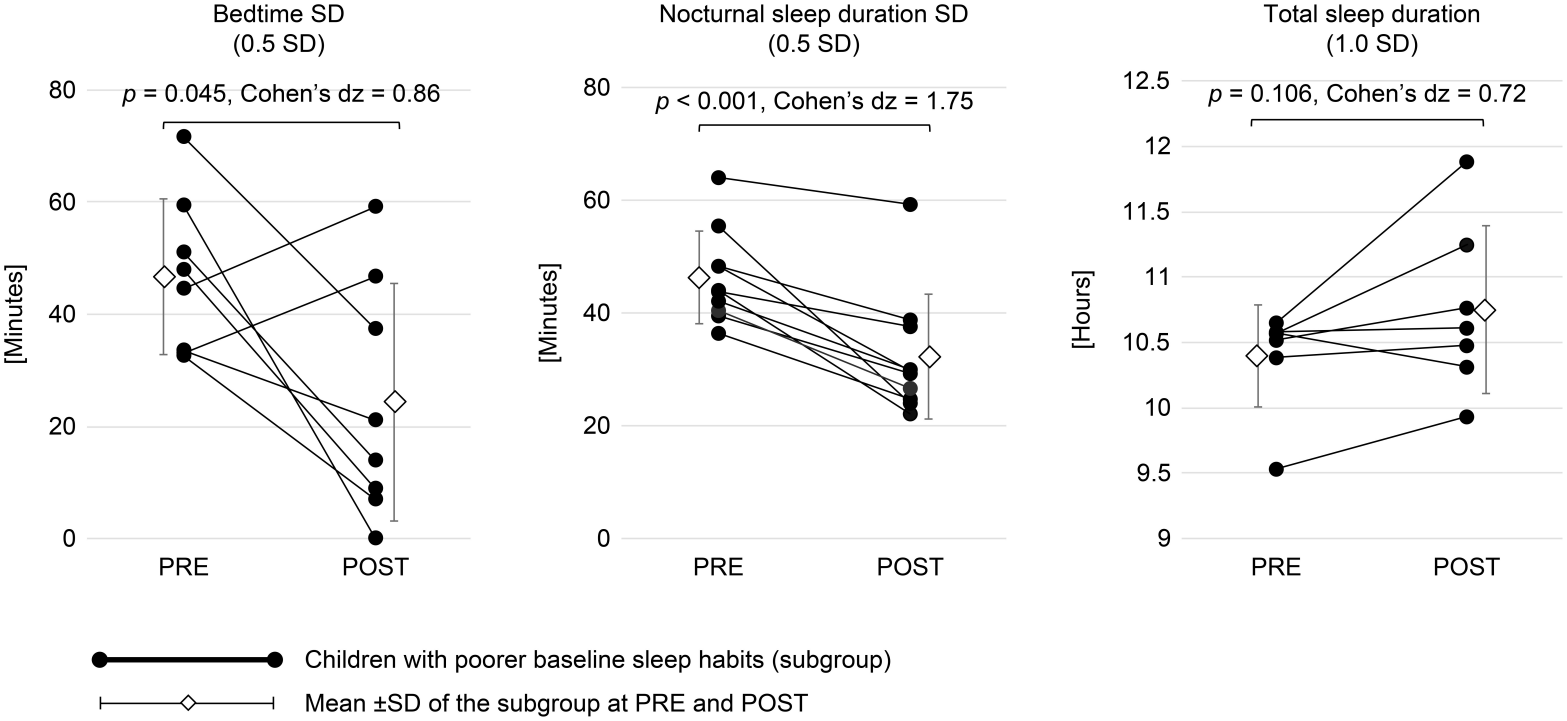
Subgroup analysis of changes in sleep outcomes among children with poorer baseline sleep habits.

Figure 3 presents subgroup analyses among children whose pre-intervention sleep parameters were more than 0.5 SD below the overall mean. Because most recruited participants generally exhibited normative sleep habits at pre-intervention, few sleep-related habits showed significant changes in the analysis of the full sample. In contrast, several additional sleep-related outcomes improved in this subgroup.

Bedtime SD significantly reduced from 46.70 ± 13.92 to 24.26 ± 21.13 min [*t*_(7)_ = −2.44, *p* = 0.045, Cohen's dz = 0.86, and *n* = 8]. Nocturnal Sleep Duration SD also decreased significantly, from 46.26 ± 8.24 to 32.27 ± 10.95 min [*t*_(9)_ = −5.52, *p* < 0.001, Cohen's dz = 1.75, and *n* = 10]. The mean number of nighttime awakenings approached significance, decreasing from 3.74 ± 1.56 to 2.10 ± 1.76, with a moderate-to-large effect size [*t*_(9)_ = −2.05, *p* = 0.070, Cohen's dz = 0.65, and *n* = 10].

Among children whose pre-intervention total sleep duration was more than 1.0 SD shorter than the overall mean (*n* = 7), total sleep duration increased from 10 h 24 min ± 23.5 min to 10 h 45 min ± 38.6 min. Although this change did not reach statistical significance, it was associated with a moderate-to-large effect size [*t*_(6)_ = 1.90, *p* = 0.106, and Cohen's dz = 0.72].

### Effects of the app on caregivers' sleep patterns and feelings about parenting

#### Quantitative outcomes

**Figure 4:**
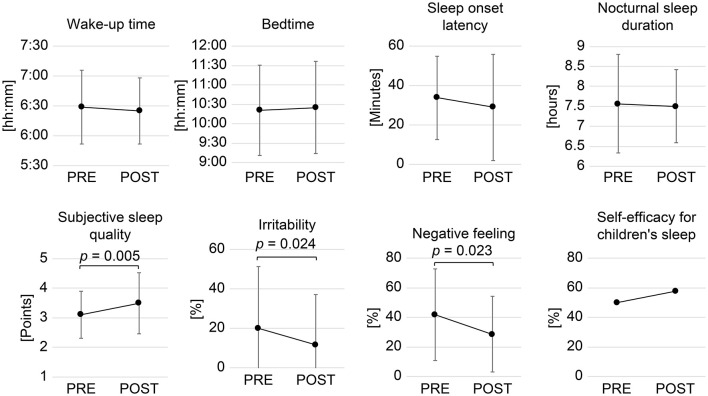
Changes in caregivers' sleep parameters, negative affect, and parenting self-efficacy.

As shown in Figure 4, caregivers' subjective sleep quality significantly improved following the intervention [pre: 3.10 ± 0.80; post: 3.49 ± 1.03; *t*_(37)_ = 2.97, and *p* = 0.005].

The proportion of days with caregiver-reported irritability while caring for their child decreased significantly [pre: 20.21 ± 31.07%; post: 11.66 ± 25.70%; *t*_(37)_ = −2.36, and *p* = 0.024]. Additionally, the proportion of days with negative feelings during parenting was also significantly reduced (pre: 41.79 ± 36.25%; post: 28.72 ± 39.53%; *t*_(37)_ = −2.37, and *p* = 0.023].

#### Quantitative feedback from the online questionnaire

Of the 50 participants enrolled, 35 (70.0%) completed the post-intervention online questionnaire regarding user experience. No formal comparison was conducted between respondents and non-respondents; however, there were no apparent differences in baseline characteristics between the two groups. Usefulness ratings showed a mean score of 3.58 (SD = 0.94). Among the 35 respondents, 17 (48.6%; scores of 4 or 5) rated the app positively.

Perceived changes in children's sleep demonstrated a mean score of 3.66 (SD 0.95). Among the 35 respondents, 21 (60.0%; scores of 4 or 5) reported positive changes in their child's sleep, 12 (34.3%) reported no change, 2 (5.7%) reported worsened sleep, and none reported somewhat worsened sleep. Among the two caregivers who reported worsened sleep, one attributed it not to the app but to the child's increased stamina from daycare initiation, which altered nap habits and led to later bedtimes. The other caregiver noted persistent night awakenings due to continued breastfeeding but acknowledged that establishing a bedtime routine with picture books facilitated smoother sleep onset.

Among the 35 respondents, changes in caregiver self-efficacy for child sleep management showed a mean score of 3.80 (SD = 0.52), with 26 (74.3%) reporting improvement (scores 4–5) and none reporting worsening. Changes in overall parenting self-efficacy had a mean of 3.57 (SD = 0.69), with 21 (60.0%) reporting improvement.

#### Qualitative feedback from semi-structured interviews

Of the 50 participants, 23 (46.0%) participated in semi-structured interviews conducted via telephone or Zoom. Participation in the interviews was voluntary, and caregivers who expressed interest were invited to schedule an interview.

##### Perceived appropriateness and human-likeness of app-delivered content

Of the 23 interview respondents, 91% (21/23) rated the advice as “always appropriate” or “mostly appropriate” to the question “Did you receive advice that was appropriate for the information you entered?” Supportive messages automatically delivered by the app were rated as “always appropriate” by 96% (22/23) of respondents.

Additionally, 78% (18/23) of the respondents rated the perceived human-likeness of the advice and comments as “very human-like” or “somewhat human-like,” with a mean rating of 4.17 (SD = 1.05). Five caregivers (22%) spontaneously indicated their belief that the advice and comments were delivered by a human, despite being informed at enrollment that the intervention was AI-based.

#### User experience and acceptability

In the post-intervention interviews, caregivers reported that the natural, human-like encouraging messages and individualized advice from the app motivated them to modify their children's behaviors. One caregiver explained, “I used to feel anxious comparing my child with information I found online, but receiving advice tailored specifically to my child helped me understand my focus, making it easier to engage with confidence.” Another caregiver remarked, “Supportive messages from the app reduced my sense of loneliness in managing parenting alone, encouraging me to continue using the app.”

Several caregivers attributed reductions in nocturnal awakenings to app-recommended behavioral changes, including adjustments to iron intake, reduced screen exposure, and consistent bedtime routines. Caregivers reporting increased self-efficacy described newfound confidence and observed positive behavioral changes. One noted, “Once I established a bedtime routine, my child started saying ‘let us read a book' when feeling sleepy. I feel my child has learned behaviors leading to sleep.” Another emphasized personalized guidance, stating, “I had been unknowingly engaging in behaviors that disrupted my child's sleep, but the app's personalized advice gave me confidence in appropriate actions.” Some caregivers reported that putting their child to sleep had become much smoother and that they no longer needed to raise their voice in the evening. Additionally, six caregivers reported that they themselves had begun sleeping better.

Caregivers reporting improved overall parenting self-efficacy described multiple perceived benefits: “Consistent sleep and wake times made it easier to plan daytime activities and reduced fussiness”; “Increased sleep duration alleviated my growth concerns for my child. With better sleep, she became more cheerful during the day, strengthening family bonds, including interactions with grandparents.”; “Earlier bedtimes gave me personal time to recover from fatigue”; “Reviewing app feedback with my husband helped us align our parenting approach”; and “The app's personalized advice was motivating, and praise for progress built my confidence in parenting.”

## Discussion

In this 6-month trial of the Nenne Navi-AI app, no formal withdrawals occurred, and 94% of caregivers (47/50) consistently entered data with ≤ 2 consecutive lapses. These findings indicate high long-term adherence and acceptability among caregivers of young children with sleep difficulties. Previous human-guided interventions with Nenne Navi^®^ reported 100% adherence over a 6-month period. This AI version maintained 94% adherence without manual support from experts, supporting the feasibility of scalable implementation.

The AI-enhanced advice selection and tailoring process employs a hybrid architecture, combining supervised machine learning and expert-derived algorithms. The algorithm achieved a precision of 0.96 in the internal validation of the beta-1 model, indicating that inappropriate recommendations were rare. These findings were consistent with the model's safety-oriented design. Qualitative user feedback indicated high acceptability, supporting the feasibility of sustained AI-enhanced sleep guidance in real-world community settings.

Giebel et al. (2025) highlighted stakeholder “perceptions of AI”—low trust, doubts about validity and benefit, and fear-based concerns—as major barriers to the adoption of AI-based clinical decision support. In contrast, caregivers in the present study rated the advice provided by the AI-enhanced system as easy to understand, contextually appropriate, and manageable in daily life. Notably, some caregivers perceived the AI-provided advice as human-delivered despite prior disclosure of its AI-based origin, suggesting a high degree of perceived naturalness. These findings in the present caregiver sample suggest that AI-related concerns may be mitigated when outputs are relevant, comprehensible, and aligned with users' routines. This perceived naturalness, along with personalized support, may have contributed to caregivers' emotional wellbeing and sustained engagement during the intervention. Interview findings further indicated that personalized feedback enhanced caregivers' confidence and emotional stability in managing sleep-related challenges.

Previous human-guided Nenne Navi^®^ interventions demonstrated significant improvements in children's wake-up times, sleep onset latency, social jetlag, night awakenings, and caregivers' parenting mood (Yoshizaki et al., 2023; Tanaka et al., 2025). In the present AI-enhanced study, significant reductions in nocturnal awakenings were observed, although improvements in wake-up times, sleep onset latency, and social jetlag were not detected. This difference may partly reflect regional characteristics of the sample, where baseline wake and bedtimes were already relatively early. National surveys indicate that residents of Aomori Prefecture aged 10 years and older have the longest average sleep duration in Japan (8 h 8 min/day) and the earliest bedtimes and wake-up times (Statistics Bureau of Japan, 2022). While total sleep duration decreased across the full sample, this likely reflects normative age-related reductions in sleep need. The observed delay in nap onset time may partly reflect age-related developmental changes in nap patterns, given the longitudinal design, and should therefore be interpreted accordingly. Subgroup analyses of children with shorter pre-intervention sleep duration showed meaningful increases with moderate-to-large effect sizes. Among children with poorer baseline sleep indices, significant reductions in variability (standard deviation) of bedtime and nocturnal sleep duration were also observed. Given previous findings linking sleep rhythm irregularity to neurodevelopmental outcomes (Iwatani et al., 2024), stabilization of sleep timing may represent a clinically meaningful change. The capacity of the AI-enhanced framework to analyze longitudinal daily input patterns and dynamically adjust recommendations may partly have contributed to these reductions in variability.

These findings suggest that the intervention may have improved sleep habits among young children. Although the magnitude of improvement did not fully reach that observed in previous human-guided interventions, the AI-enhanced system achieved meaningful behavioral changes without clinician involvement, supporting the feasibility of delivering structured behavioral sleep support at scale.

In addition to child sleep outcomes, caregivers reported significant improvements in sleep quality, irritability, and negative emotional states. Given the bidirectional relationship between child sleep and caregiver wellbeing, these findings suggest that AI-enhanced sleep guidance may contribute to broader family-level support rather than solely child-focused outcomes.

Most existing AI tools in sleep medicine primarily focus on physiological monitoring, such as sleep staging and respiratory event detection (Piccini et al., 2023; May and Dalton, 2024). Unlike these approaches, hybrid clinical decision systems integrating rule-based reasoning with machine learning have been explored to enhance transparency and safety while maintaining predictive performance (Kierner et al., 2025). The present framework adopts a similar hybrid strategy by integrating expert-defined behavioral rules within a supervised machine learning architecture. Rather than replacing expert judgment, this hybrid system operationalizes clinical expertise within a safety-oriented algorithmic structure. By combining adaptive inference with predefined rule-based safeguards, the system enables personalized behavioral guidance while maintaining strict safety thresholds.

In summary, this study provides preliminary evidence supporting the feasibility of a culturally adapted, safety-oriented hybrid AI framework for delivering behavioral sleep and parenting support in community settings. Further randomized controlled trials are warranted to determine long-term efficacy and developmental outcomes.

## Limitations

This study has several limitations. First, it was a pilot study focused on long-term adherence and feasibility conducted in a community setting with minimal eligibility restrictions. The absence of a waitlist control group precludes ruling out regression to the mean (Barnett et al., 2005). Because the study relied on pre–post within-subject comparisons, the observed improvements may partly reflect within-subject variability or temporal trends rather than intervention effects alone. Additionally, the relatively small sample size (*n* = 50) warrants caution regarding the generalizability of the findings.

Incentives provided to caregivers may have reduced attrition. While we attempted to minimize this influence by offering incentives before the intervention rather than contingent upon its completion, we cannot fully exclude the possibility that the incentives impacted continued participation.

Study participants in this region exhibited earlier average bedtimes and wake times, possibly due to local cultural or environmental factors, which may have limited the observed intervention effects. Although the hybrid AI system demonstrated high precision, its performance was evaluated within a specific cultural and caregiving context. In addition, the training dataset for the AI model included only maternal responses, potentially limiting the generalizability of the findings to families in which fathers are the primary caregivers. Further validation in larger and more diverse populations, including those with broader sociodemographic and caregiving backgrounds, is necessary.

We relied on caregiver-reported sleep habit data collected via the app, which may be subject to reporting bias. While prior validation using actigraphy supports the accuracy of children's sleep rhythm measures obtained through the application (Yoshizaki et al., 2020), some degree of reporting bias cannot be excluded. Future studies should continue to refine measurement strategies and, where feasible, complement app-based reports with objective or externally validated indicators to further enhance measurement validity. Moreover, some outcome measures were based on pragmatic, study-specific indicators rather than on those of fully standardized and validated instruments, which may limit comparability across studies. Therefore, future research should incorporate standardized and validated assessment tools.

Due to the pilot design (*n* = 50, no control group), potential biases (incentives, caregiver reports), and regional participant characteristics, this study may lack the statistical power to detect all intervention effects. Nevertheless, preliminary information on clinically meaningful outcomes was obtained, demonstrating high feasibility and acceptability of the intervention. These findings will inform the design of future large-scale studies. Furthermore, improving the transparency and explainability of AI-enhanced recommendations is important for building user trust and clinical applicability.

## Conclusion

In this community-based pilot study, the AI-enhanced sleep support system Nenne Navi-AI demonstrated high feasibility, with excellent 6-month adherence and favorable usability feedback. The system shows promise for improving sleep habits in early childhood, enhancing caregiving experiences, and reducing parenting stress. However, this pilot study was conducted in a single community with a relatively small sample size, which may limit its generalizability. These findings support the potential of scalable AI-assisted approaches for pediatric sleep support and warrant further evaluation in larger controlled studies.

## Data Availability

The data supporting the findings of this study are not publicly available because they contain information that could compromise the privacy of pediatric research participants. Requests to access the datasets should be directed to AY, arika@kokoro.med.osaka-u.ac.jp.

## References

[B1] Al Mahmud A. Wu J. Mubin O. (2022). A scoping review of mobile apps for sleep management: user needs and design considerations. Front. Psychiatry 13:1037927. doi: 10.3389/fpsyt.2022.1037927 36329917 PMC9624283

[B2] An Q. Rahman S. Zhou J. Kang J. J. (2023). A comprehensive review on machine learning in healthcare industry: classification, restrictions, opportunities and challenges. Sensors (Basel). 23:4178. doi: 10.3390/s23094178 37177382 PMC10180678

[B3] Ananth S. (2021). Sleep apps: current limitations and challenges. Sleep Sci. 14:83–86. doi: 10.5935/1984-0063.20200097 34104344 PMC8157780

[B4] Barnett A. G. van der Pols J. C. Dobson A. J. (2005). Regression to the mean: what it is and how to deal with it. *Int. J. Epidemiol*. 34, 215–220. doi: 10.1093/ije/dyh299 15333621

[B5] Bathory E. Tomopoulos S. (2017). Sleep regulation, physiology and development, sleep duration and patterns, and sleep hygiene in infants, toddlers, and preschool-age children. Curr. Probl. Pediatr. Adolesc. Health Care 47, 29–42. doi: 10.1016/j.cppeds.2016.12.001 28117135

[B6] Cohen J. (1988). Statistical Power Analysis for the Behavioral Sciences, 2nd Edn. Hillsdale, NJ: Lawrence Erlbaum Associates.

[B7] Dai Y. Zhang L. Li X. Chen H. Wang J. (2026). Usability, acceptability and future opportunities of mobile health (mHealth) apps for caregiver health decision-making for children: a scoping review. BMC Digit. Health 1:35. doi: 10.1186/s44247-026-00235-2

[B8] Finkel M. A. Duong N. Hernandez A. Goldsmith J. Rifas-Shiman S. L. Dumitriu D. . (2024). Associations of infant sleep characteristics with childhood cognitive outcomes. J. Dev. Behav. Pediatr. 45, e560–e568. doi: 10.1097/DBP.0000000000001311 39140879 PMC11645234

[B9] Giebel G. D. Raszke P. Nowak H. Palmowski L. Adamzik M. Heinz P. . (2025). Problems and barriers related to the use of AI-based clinical decision support systems: interview study. J. Med. Internet Res. 27:e63377. doi: 10.2196/63377 39899342 PMC11833262

[B10] Gkintoni E. Vassilopoulos S. P. Nikolaou G. Boutsinas B. (2025). Digital and AI-enhanced cognitive behavioral therapy for insomnia: neurocognitive mechanisms and clinical outcomes. J. Clin. Med. 14:2265. doi: 10.3390/jcm14072265 40217715 PMC11989647

[B11] Hiscock H. Cook F. Bayer J. Le H. N. Mensah F. Cann W. . (2014). Preventing early infant sleep and crying problems and postnatal depression: a randomized trial. Pediatrics 133, e346–e354. doi: 10.1542/peds.2013-1886 24394682

[B12] Holzinger A. Biemann C. Pattichis C. S. Kell D. B. (2017). What do we need to build explainable AI systems for the medical domain? *arXiv* [Preprint]. *arXiv:1712.09923*. Available online at: https://arxiv.org/abs/1712.09923 (Accessed March 1, 2026).

[B13] Huhdanpää H. Morales-Muñoz I. Aronen E. T. Pölkki P. Saarenpää-Heikkilä O. Paunio T. . (2019). Sleep difficulties in infancy are associated with symptoms of inattention and hyperactivity at the age of 5 years: a longitudinal study. J. Dev. Behav. Pediatr. 40, 432–440. doi: 10.1097/DBP.0000000000000684 31166249 PMC6738636

[B14] Iwatani Y. Kagitani-Shimono K. Ono A. Yamamoto T. Mohri I. Yoshizaki A. . (2024). Regular sleep habits in toddlers are associated with social development and brain coherence. Sleep Med. 124, 531–539. doi: 10.1016/j.sleep.2024.10.018 39447527

[B15] Jiang F. Jiang Y. Zhi H. Dong Y. Li H. Ma S. . (2017). Artificial intelligence in healthcare: past, present and future. Stroke Vasc. Neurol. 2, 230–243. doi: 10.1136/svn-2017-000101 29507784 PMC5829945

[B16] Kierner S. Kierner P. Kucharski J. (2025). Combining machine learning models and rule engines in clinical decision systems: exploring optimal aggregation methods for vaccine hesitancy prediction. Comput. Biol. Med. 188:109749. doi: 10.1016/j.compbiomed.2025.109749 39983355

[B17] Kohyama J. Shiiki T. Ohinata-Sugimoto J. S. Hasegawa T. (2002). Potentially harmful sleep habits of 3-year-old children in Japan. J. Dev. Behav. Pediatr. 23, 67–70. doi: 10.1097/00004703-200204000-00001 11943967

[B18] Kuki A. Terui A. Sakamoto Y. Osato A. Mikami T. Nakamura K. . (2024). Prevalence and factors of sleep problems among Japanese children: a population-based study. Front. Pediatr. 12:1332723. doi: 10.3389/fped.2024.1332723 38638584 PMC11024267

[B19] Leichman E. S. Gould R. A. Williamson A. A. Walters R. M. Mindell J. A. (2020). Effectiveness of an mHealth intervention for infant sleep disturbances. Behav. Ther. 51, 548–558. doi: 10.1016/j.beth.2019.12.011 32586429 PMC7428151

[B20] Liu J. Ji X. Pitt S. Wang G. Rovit E. Lipman T. . (2024). Childhood sleep: physical, cognitive, and behavioral consequences and implications. World J. Pediatr. 20, 122–132. doi: 10.1007/s12519-022-00647-w 36418660 PMC9685105

[B21] Lokhandwala S. Spencer R. M. C. (2022). Relations between sleep patterns early in life and brain development: a review. Dev. Cogn. Neurosci. 56:101130. doi: 10.1016/j.dcn.2022.101130 35779333 PMC9254005

[B22] Lu Y. A. Lin H. C. Tsai P. S. (2025). Effects of digital sleep interventions on sleep among college students and young adults: systematic review and meta-analysis. J. Med. Internet Res. 27:e69657. doi: 10.2196/69657 40354636 PMC12107209

[B23] Matthews K. A. Pantesco E. J. (2016). Sleep characteristics and cardiovascular risk in children and adolescents: an enumerative review. Sleep Med. 18, 36–49. doi: 10.1016/j.sleep.2015.06.004 26459685 PMC4689674

[B24] May A. M. Dalton J. E. (2024). Comparison of machine learning approaches for positive airway pressure adherence prediction in a veteran cohort. Front Sleep. 3:1278086. doi: 10.3389/frsle.2024.1278086 41424513 PMC12713837

[B25] Mindell J. A. Du Mond C. E. Sadeh A. Telofski L. S. Kulkarni N. Gunn E. (2011). Efficacy of an internet-based intervention for infant and toddler sleep disturbances. Sleep 34, 451–458. doi: 10.1093/sleep/34.4.451 21461323 PMC3065255

[B26] Mindell J. A. Sadeh A. Wiegand B. How T. H. Goh D. Y. (2010). Cross-cultural differences in infant and toddler sleep. Sleep Med. 11, 274–280. doi: 10.1016/j.sleep.2009.04.012 20138578

[B27] Morales-Muñoz I. Broome M. R. Marwaha S. (2020). Association of parent-reported sleep problems in early childhood with psychotic and borderline personality disorder symptoms in adolescence. JAMA Psychiatry 77, 1256–1265. doi: 10.1001/jamapsychiatry.2020.1875 32609357 PMC7330826

[B28] Murata E. Yoshizaki A. Fujisawa T. X. Tachibana M. Taniike M. Mohri I. (2023). What daily factors affect the sleep habits of Japanese toddlers? J. Clin. Sleep Med. 19, 1089–1101. doi: 10.5664/jcsm.10508 36789883 PMC10235708

[B29] Norman G. R. Sloan J. A. Wyrwich K. W. (2003). Interpretation of changes in health-related quality of life: the remarkable universality of half a standard deviation. Med. Care 41, 582–592. doi: 10.1097/01.MLR.0000062554.74615.4C 12719681

[B30] Park J. Jeon H. Choi E. K. (2024). Digital health intervention on patient safety for children and parents: a scoping review. J. Adv. Nurs. 80, 1750–1760. doi: 10.1111/jan.15954 37950382

[B31] Piccini J. August E. Óskarsdóttir M. Arnardóttir E. S. (2023). Using the electrodermal activity signal and machine learning for diagnosing sleep. Front. Sleep 2:1127697. doi: 10.3389/frsle.2023.1127697 41426452 PMC12713860

[B32] Revicki D. Hays R. D. Cella D. Sloan J. (2008). Recommended methods for determining responsiveness and minimally important differences for patient-reported outcomes. J. Clin. Epidemiol. 61, 102–109. doi: 10.1016/j.jclinepi.2007.03.012 18177782

[B33] Sato M. Kanikowska D. (2025). Difference of sleep time and screen time in preschool children in rural and urban settings in natural living conditions in Japan. Int. J. Biometeorol. 69, 1487–1493. doi: 10.1007/s00484-025-02906-7 40198346

[B34] Sivertsen B. Harvey A. G. Reichborn-Kjennerud T. Torgersen L. Ystrom E. Hysing M. (2015). Later emotional and behavioral problems associated with sleep problems in toddlers: a longitudinal study. JAMA Pediatr. 169, 575–582. doi: 10.1001/jamapediatrics.2015.0187 25867179

[B35] Sivertsen B. Harvey A. G. Reichborn-Kjennerud T. Ystrom E. Hysing M. (2021). Sleep problems and depressive symptoms in toddlers and 8-year-old children: a longitudinal study. J. Sleep Res. 30:e13150. doi: 10.1111/jsr.13150 32743857

[B36] Statistics Bureau of Japan (2022). 2021 Survey on Time Use and Leisure Activities [in Japanese]. Availble online at: https://www.stat.go.jp/data/shakai/2021/kekka.html (accessed January 15, 2026).

[B37] Su P. Taniike M. Ohno Y. Mohri I. (2024). Are the sleep–wake cycle and sleep duration ethnically determined? A comparison of Tibetan and Japanese children's sleep. Clocks Sleep 6, 682–689. doi: 10.3390/clockssleep6040046 39584975 PMC11586942

[B38] Tanaka S. Yoshizaki A. Fujisawa T. X. Murata E. Kosaka T. Shinkawa H. . (2025). Improving children's sleep habits using an interactive smartphone app: effect on children's sleep and caregiver. Sleep Med X. 10:100156. doi: 10.1016/j.sleepx.2025.100156 41209544 PMC12593587

[B39] Verma R. K. Dhillon G. Grewal H. Prasad V. Munjal R. S. Sharma P. . (2023). Artificial intelligence in sleep medicine: present and future. World J. Clin. Cases 11, 8106–8110. doi: 10.12998/wjcc.v11.i34.8106 38130791 PMC10731177

[B40] Wang S. H. Lin K. L. Chen C. L. Chiou H. Chang C. J. Chen P. H. . (2024). Sleep problems during early and late infancy: diverse impacts on child development trajectories across multiple domains. Sleep Med. 115, 177–186. doi: 10.1016/j.sleep.2024.02.018 38367360

[B41] Yimenu T. K. Adege A. B. Techan S. Y. (2025). Integrating expert knowledge with machine learning for AI-based stroke identifications and treatment systems. Digit. Health 11:20552076251336853. doi: 10.1177/20552076251336853 40321889 PMC12048753

[B42] Yoshizaki A. Mohri I. Yamamoto T. Shirota A. Okada S. Murata E. . (2020). An interactive smartphone app, Nenne Navi, for improving children's sleep: pilot usability study. JMIR Pediatr. Parent. 3:e22102. doi: 10.2196/22102 33122163 PMC7738258

[B43] Yoshizaki A. Murata E. Yamamoto T. Fujisawa T. X. Hanaie R. Hirata I. . (2023). Improving children's sleep habits using an interactive smartphone app: community-based intervention study. JMIR mHealth uHealth 11:e40836. doi: 10.2196/40836 36641237 PMC9960041

